# Age Equity: A Framework for Addressing Ageism, Stigma, and Bias

**DOI:** 10.1093/geroni/igab046.1667

**Published:** 2021-12-17

**Authors:** Charles Emlet, Karen Fredriksen Goldsen

**Affiliations:** 1 University of Washington, Tacoma, Washington, United States; 2 University of Washington, Seattle, Washington, United States

## Abstract

In the Covid-19 context, researchers and policy makers have turned their attention to long-standing disparities in health equity, including by race, ethnicity, poverty, sexuality, and gender. Yet, scholarship to date does not conceptualize age as a critical aspect of difference requiring an equity lens. In this presentation, we utilize an Age Equity Framework to investigate ageism based on research findings from the 2018 National Health, Aging and Sexuality/Gender Study (NHAS): Aging with Pride. Investigating ageism, stigma, and bias, we found nearly half of LGBTQ older adults feel invisible and disrespected. After adjusting for background characteristics, experiences of ageism were associated with higher rates of stigma, lifetime victimization, discrimination, lower support and community engagement, and adverse outcomes (lower mental and physical health and quality of life). The rapidly growing older adult population highlights the pressing need to consider age inequities and the importance of achieving age equity across the life course.

